# Cancrum Oris

**Published:** 1860-11

**Authors:** Charles Brackett

**Affiliations:** Rochester, Ind.


### ARTIC E 3.

### CANCRUM ORIS.

BY CHARLES BRACKETT, M. D., ROCHESTER, IND.

Hoffman, aged fourteen months, of a weak habit, and lymphatic temperament; the child of a diseased mother, presented, on the 7th of July, 1860, at 10 o'clock A. M., the following appearances:

A small, dark spot under the right ala nasi, a thin, acrid discharge from both nostrils, some little salivary discharge mixed with thin blood, a bright redness of the whole upper lip, on the right side, reaching nearly to the center of the cheek, the redness accompanied by a swelling of the tissues of a stony hardness, so far as the redness extened; internally the lip, opposite the dark spot under the ala nasi ulcerated, of dark, dirty red appearance.

Abdomen puffed but flabby, lungs sound, eyes bright, pulse 160, appetite good, urine pale, bowels rather loose, stools offensive. I told the parents that the disease was black canker; would probably destroy the tissues so far as they were red and hard, but that life might possibly be saved.

They had been using several washes, such as acetat. plumbi. sul cupri, but all irritants used, only added to the rapid spread of the disease. At 7 P. M. of that day, a piece of the lip, its whole depth, and as broad as my fore-finger, was black and sloughed out. Teeth and gums not affected. The disease thence spread as far as the limits I had assigned it laterally, and upward so as to take a small portion of the right ala, and septum nasi till the evening of July 19th, when it died from exhaustion. No post mortem made.

I treated the disease locally only with mild unirritating washes, believing that the few fatal cases of this disease which I saw in 1851, and reported in the May number of the *Northwestern Medical and Surgical Journal*, at this stage were rendered more rapid by the various caustic and irritating applications then used, or at least were not benefited by them. Internally I gave quinine and wine, with small doses of iodid. potas., morphia and soda, or carb. ammonia, as necessary to relieve pain, also using daily small doses of ext. belladonna. Phosphates of iron and lime were also given with a stimulating, nourishing and easily digestible diet. Urine, after a few days, became dark, and was passed with much pain. On the 14th I allowed them to use a wash of sul. cupri, alum, and honey, it had, however, no perceptible effect.

A sister of the deceased, on the 17th July, had a swollen upper lip, which was abraded on the mucous surface, and of a bright red color. I touched it twice with nit. argt. on the 17th, and all disease vanished.

I am forced to believe that no treatment will stop the gangrene and erosion, after the parts have become partly disorganized by a few days of the stony hard swelling preceding the actual gangrene. During the first few days the disease may be easily subdued, but after that I believe, with most others, that it is very little under the control of remedies.

This child had previously had several attacks of sore mouth in some of its various forms, which had been easily subdued by Dr. Collins, of Bloomingsburgh, Fulton county, where the parents reside.

I have had an unusual number of cases of sore mouth this season, but none troublesome in the way of cure.

---

ARTIC E 4.

